# Hypoxia-elicited Exosomes Promote the Chemoresistance of Pancreatic Cancer Cells by Transferring LncROR via Hippo Signaling

**DOI:** 10.7150/jca.81320

**Published:** 2023-04-17

**Authors:** Huizhi Wang, Jingyu Min, Chunhui Xu, Yawen Liu, Zhengyue Yu, Aihua Gong, Min Xu

**Affiliations:** 1 Department of Gastroenterology, Affiliated Hospital of Jiangsu University, Jiangsu University, 438 Jiefang Road, Zhenjiang 212001, China; 2 Department of Gastroenterology, Changshu No.2 People's Hospital, 68 Haiyu South Road, Changshu 215500, China; 3 Department of Cell Biology, School of Medicine, Jiangsu University, 301 Xuefu Road, Zhenjiang 212013, China

**Keywords:** Exosomes, hypoxia, stemness, chemoresistance, lncROR, pancreatic cancer

## Abstract

Recent studies have found that hypoxia contributes to tumor progression and drug resistance by inducing the secretion of exosomes. However, the mechanism underlying this resistance in pancreatic cancer remains to be explored. In this study, we investigated the effect of hypoxia-induced tumor-derived exosomes (Hexo) on stemness and resistance to gemcitabine in pancreatic cancer cells, as well as the molecular mechanisms involved in this process. Firstly, we discovered that hypoxia promoted stemness and induced resistance to gemcitabine in pancreatic cancer cells. Secondly, we showed that exosomes secreted by pancreatic cancer cells under normoxic or hypoxic conditions can be transfected into tumor cells. Thirdly, it was demonstrated that Hexo promotes proliferation, stemness, and resistance to gemcitabine in pancreatic cancer cells, as well as inhibits the apoptosis and cell cycle arrest induced by gemcitabine. Finally, it was verified that Hexo inactivated the Hippo/Yes-associated protein (Hippo/YAP) pathway in pancreatic cancer cells by transferring exosomal long non-coding RNA regulator of reprogramming (lncROR). In summary, the hypoxic tumor microenvironment could promote stemness and induce resistance to gemcitabine in pancreatic cancer cells. Mechanistically, Hexo enhanced stemness to promote chemoresistance in pancreatic cancer cells by transferring lncROR via Hippo signaling. Thus, exosomal lncROR may serve as a candidate target of chemotherapy for pancreatic cancer.

## Introduction

Pancreatic cancer (PC) is an aggressive malignancy and the fourth leading cause of cancer-related death 1. The 5-year survival rate of patients with PC is <10% due to the lack of typical early symptoms 1. Despite developments in targeted therapy and immunotherapy, drug resistance remains a major challenge in the treatment of PC 2. In the past decade, gemcitabine has been widely used as a first-line chemotherapy drug for PC. However, the efficacy of gemcitabine is poor because PC cells are resistant to this agent. Therefore, it is of great importance to explore the mechanism of resistance to gemcitabine in PC.

Exosomes are small (diameter: 30-150 nm) extracellular vesicles with a bilayer lipid membrane secreted by most cells. These vesicles contain various biomolecules, such as microRNAs (miRNAs) and proteins 3. It is established that exosomes are involved in diverse functions as messengers in cell-to-cell communication, particularly in carcinogenesis 4. Moreover, a number of studies have found that exosomes participate in the regulation of tumor migration, invasion and angiogenesis, as well as affect various biological processes (e.g., tumor immune escape and chemoresistance) in different types of tumors (e.g., breast 5, lung 6, prostate 7, and colon 8). In PC, Richards et al. found that exosomes secreted by cancer-associated fibroblasts could regulate cell proliferation and confer resistance to gemcitabine through miRNAs targeting phosphatase and tensin homolog (PTEN) 9. Additionally, exosomes with downregulated hsa_circ_0000069 could suppress the malignant transformation of human pancreatic duct epithelial cells 10.

Numerous studies demonstrated that exosomes play a role as critical mediators of the composition of the tumor microenvironment (TME) which consists of blood vessels, extracellular matrix, cytokines, stromal cells (tumor-associated fibroblasts, endothelial cells, immune cells, and mesenchymal stem cells, etc.), and the chemical microenvironment (pH, oxygen, nitric oxide, metabolites, etc.) 11. Previous studies indicated that the TME may mediate drug resistance through multiple mechanisms, including the prevention of immune clearance of tumor cells, hindrance of drug uptake, and expression of paracrine growth factors to stimulate cancer cell growth 11. Additionally, it was found that the concentration of oxygen near the perfusion vessels was slightly higher than that measured far from those vessels 12. Tumor cells located far from the perfusion vessels exist in a relatively hypoxic TME 12. The increased expression of hypoxia-inducible factor 1α (HIF-1α) is a key marker for a hypoxic TME 12. A hypoxic TME has been associated with tumor proliferation. For example, hypoxic glioma stem cell-derived exosomal Linc01060 promoted glioma progression through the myeloid zinc finger 1/c-Myc/HIF-1α (MZF1/c-Myc/HIF-1α) axis 13. Furthermore, in ovarian cancer, hypoxia-induced exosomes contributed to tumor aggressiveness and chemoresistance 14. In PC, hypoxic derived exosomal miR-30b-5p promoted angiogenesis by inhibiting gap junction protein alpha 1 (GJA1) 15, while miR-301a mediated M2 macrophage polarization to promote metastasis via PTEN/phosphoinositide 3-kinase-γ (PTEN/PI3Kγ) 16. However, few studies have attempted to elucidate the mechanism of hypoxic exosomes underlying the development of resistance to chemotherapy in PC. A study revealed that hypoxic exosomal circular RNA zinc finger protein 91 (circZNF91) promoted chemoresistance in normoxic PC cells by enhancing glycolysis 17. In addition, exosomal long non-coding RNA regulator of reprogramming (lncROR) promoted tumor growth in PC, and lncROR enriched in exosomes was secreted by PC cells 18. Therefore, we hypothesized that a hypoxic TME stimulates the secretion of more exosomes by tumor cells to induce resistance to gemcitabine in normoxic tumor cells via lncROR.

In this study, we aimed to identify the impact of hypoxia induced tumor-derived exosomes (Hexo) on the stemness and gemcitabine resistance in pancreatic cancer cells, and discover the mechanism underlying the chemoresistance. Our findings provide evidence for that exosomal lncROR may serve as a candidate target for pancreatic cancer chemotherapy.

## Materials and methods

### Cell culture

Human PANC1 and PaTu8988 PC cell lines, the immortalized pancreatic ductal epithelial cell line H6C7, and human embryonic kidney cell line HEK293T were obtained from the American Type Culture Collection (Manassas, VA, USA). All cells were tested and authenticated by short tandem repeat analysis. Cells were cultured in Dulbecco's modified Eagle's medium (DMEM; HyClone, Beijing, China) containing 10% fetal bovine serum and 1% penicillin and streptomycin (Hyclone, South Logan, UT, USA) in a humidified incubator at 37°C and 5% CO_2_ under hypoxic (1% O_2_) or normal (21% O_2_) conditions. Plates with adherent cells were placed in the anoxic culture box, which was filled with the premixed hypoxic mixed gas (1% O_2_, 5% CO_2_, and 94% N_2_) for approximately 5 min. An appropriate amount of petroleum jelly liquid was applied to the seal to ensure the airtightness of the anoxic box. Subsequently, the anoxic box was placed in the cell culture incubator at 37℃.

### Antibodies and chemicals

The antibodies used in this study were rabbit anti-HIF-1α (CAT 3716; Cell Signaling Technology [CST], Danvers, MA, USA), rabbit anti-octamer-binding transcription factor 4 (anti-OCT4; CAT 2750; CST), rabbit anti-NANOG (CAT 4903; CST), rabbit anti-SRY-box transcription factor 2 (anti-SOX2; CAT 3579; CST), mouse anti-CD44 (CAT 5640; CST), rabbit anti-CD133 (CAT 64326; CST), rabbit anti-β-tubulin (CAT 21058; Abcam), rabbit anti-CD9 (CAT 13174; CST), rabbit anti-CD63 (CAT 68418, Abcam), rabbit anti-CD81 (CAT 56039; CST), rabbit anti-Bcl2 associated X (anti-Bax; CAT 2772; CST), rabbit anti- B-cell CLL/lymphoma 2 (anti-Bcl-2; CAT 4223; CST), mouse anti-caspase-3 (anti-CASP3; CAT 9668; CST), and mouse anti-CASP9 (CAT 9504; CST). The Hippo Signaling Antibody Sampler Kit (CAT 8579) was obtained from CST, and gemcitabine (HY-17026) was obtained from MedChem Express (Princeton, NJ, USA).

### Western blotting

Cells were rinsed with cold phosphate-buffered saline (PBS), and lysed with radioimmunoprecipitation assay buffer containing 1% phenylmethylsulfonyl fluoride, 1% protease inhibitor, 5% 2‐mercaptoethanol, and 93% 2 × loading buffer. The cell lysates were boiled at 100°C for 10 min and centrifuged at 12,000 × *g* for 10 min. Protein concentration was determined using the bicinchoninic acid assay. Subsequently, total protein samples were separated by sodium dodecyl sulfate-polyacrylamide gel electrophoresis and transferred onto polyvinylidene difluoride membranes for immunoblotting assays. Membranes were blocked with 5% bovine serum albumin for 1 h at room temperature and incubated with primary antibodies overnight at 4°C. Thereafter, the membranes were washed and incubated with the respective horseradish peroxidase-conjugated secondary antibody for 1 h at room temperature. Finally, proteins were visualized with the ECL detection system (Amersham Pharmacia Biotech, Little Chalfont, UK).

### Cell Counting Kit-8 assay

Cells (4×10^3^ per 100 μL) were seeded into 96-well plates, cultured for 24 h, and treated with gemcitabine (0.15, 0.3, 0.6, 1.2, 2.4, 1, 2, 4, 8, 16 μM) for 72 h. After removing the supernatant, cells were incubated with Cell Counting Kit-8 reagent (10 μL) in the dark for 2 h at 37°C. The absorbance at 450 nm was measured using a microplate reader (Bio-Rad Laboratories, Hercules, CA, USA). Each experiment was performed in triplicate, and the results were analyzed using GraphPad Prism version 7 software (GraphPad Software Inc., San Diego, CA, USA).

### Quantitative real-time polymerase chain reaction (qRT-PCR)

Total RNA was extracted via RNAiso Plus (Invitrogen, Carlsbad, CA, USA) according to the instructions provided by the manufacturer. A RevertAid First Strand cDNA Synthesis Kit (Thermo Scientific) was utilized for the synthesis of complementary DNA from RNA samples (1 μg). A SYBR Green Mix kit (Bio Rad Laboratories) was used to perform quantitative real-time polymerase chain reaction. The relative expression was determined based on the 2^-ΔΔCt^ method. The primers used in this experiment are presented in Table 1.

### Exosome isolation and purification

Exosomes were isolated using an exosome extraction kit (EXOTC50A-1; System Biosciences, Palo Alto, CA, USA). In brief, cells were cultured in DMEM containing 10% exosome‑depleted fetal bovine serum (FBS) (subjected to 100,000 × *g* ultracentrifugation for 16 h). The conditioned medium was collected through 300 × *g* centrifugation for 10 min after treatment for 48 h. Subsequently, the supernatant was centrifuged at 2,000 × *g* for 30 min and at 12,000 × *g* for 30 min. Thereafter, exosomes were extracted from the supernatant according to instructions provided by the manufacturer. Exosomes were resuspended in PBS, purified through a 0.22-μm filter, and analyzed by transmission electron microscopy (TEM). Exosome size distribution was calculated using nanoparticle tracking analysis (NTA). In addition, the levels of exosome-specific proteins (i.e., CD9, CD63, and CD81) were detected via western blotting. The concentration of exosomes was evaluated using a bicinchoninic acid protein kit (Beyotime, Shanghai, China). For exosome treatment, the cells were cultured with purified exosomes at 100 μg/mL, unless otherwise specified.

### Exosome labeling and tracking

Exosomes were labeled using the dioctadecyloxacarbocyanine (Dio) Green Fluorescent membrane linker dye (Thermo, USA) according to the instructions provided by the manufacturer. Next, for the exosome uptake assay, the traced exosomes were incubated with PANC1 cells for 48 h at 37°C with 5% CO_2_. Subsequently, PBS was used to wash the cells, and 4′, 6-diamidino-2-phenylindole (Leagene, Beijing, China) was used to stain nuclei for 10 min at room temperature. Finally, images were captured using an Axio-Imager-LSM800 laser scanning microscope (ZEISS, Oberkohen, Germany). The experiment was performed in the dark.

### Colony formation assay

Cells were treated with PBS, normoxic exosomes (Nexo) or hypoxia induced tumor-derived exosomes (Hexo) for 48 h. Thereafter, cells (1×10^3^ per well) were seeded in six-well plates and incubated at 37°C and 5% CO_2_ for 10-14 days. Finally, colonies were fixed in 4% paraformaldehyde and stained with 0.5% crystal violet (Leagene) for 30 min. The number of colonies formed was counted using the ImageJ software (National Institutes of Health, Bethesda, MD, USA).

### Sphere formation assay

PANC1 cells (2×103 per well) co-cultured with exosomes for 48 h were seeded into six-well plates containing serum-free DMEM-F12 supplemented with 20 ng/mL epidermal growth factor (EGF; Beyotime), 20 ng/mL basic fibroblast growth factor (bFGF; Beyotime), and 1 × B27 (Gibco, USA). The plates were maintained at 37°C and 5% CO2 until the formation of spheroids. Finally, the number of spheres with size > 50 μm was determined and plotted.

### Apoptosis Assay

Firstly, PANC1 cells were treated with PBS, Nexo, or Hexo for 48 h. Secondly, 1×10^5^ cells were co-cultured with PBS, Nexo (200 μg/well), or Hexo (200 μg/well) in six-well plates containing DMEM supplemented with 10% exosome-free FBS for 24 h. Thirdly, gemcitabine at the concentration of 16 μM was added to the plates, and the cells were incubated for 72 h. Next, 1×10^5^ cells were counted and stained with the Annexin V-APC/PI kit (Fcmacs, Nanjing, China) according to the instructions provided by the manufacturer. Finally, the presence of apoptotic cells was examined using Fortessa FACS (BD Biosciences, Lake Franklin, NJ, USA), and the apoptosis rate was calculated using the FlowJo software 7.6 (BD Biosciences).

### Cell Cycle Analysis

Cells (8×10^5^) treated with PBS, 100 μg/mL Nexo, or 100 μg/mL Hexo for 48 h were co-cultured with PBS, Nexo (200 μg/well), or Hexo (200 μg/well) in DMEM containing 10% exosome-free FBS in six-well plates for 24 h. Subsequently, PBS or 16 μM gemcitabine was added to the plates for 72 h in the dark. Cells were harvested and fixed in cold 70% ethanol for 24 h. Thereafter, cells were stained with the Cell Cycle Staining Kit (MultiSciences, Hangzhou, China) according to the instructions provided by the manufacturer. Samples were evaluated with Fortessa FACS (BD Biosciences), and the Modfit LT 5.0 software (Verity Software House, Topsham, ME, USA) was used to analyze cell cycle distribution.

### Cell transfection and viral infection

The plasmids pCDH RoR and pCDH Vector were provided by the Basic Medical Science Institute, Jiangsu University (Zhenjiang, China). DNA sequencing was used to confirm the sequence. Transient transfection was performed using Lipfectamine2000 reagent (Invitrogen) according to the instructions provided by the manufacturer. Plasmid shNC or shROR was co-transfected with psPAX2 and pMD2.G into 293T cells for 48 h and 72 h. Viral particles were collected from the supernatant following centrifugation of cells. PANC1 cells were infected with the virus in DMEM containing polybrene (8 mg/mL; Sigma-Aldrich). After 48 h, the culture medium, which included puromycin, was replaced to select infected cells.

### Statistical analysis

Each experiment was performed separately at least three times. The data was presented as the mean ± standard deviation (SD). Student's t-test determined the statistical significance between the two groups, while a two-way analysis of variance (ANOVA) was used to analyze the data more than 3 groups. GraphPad Prism 8 software was used to present the results. The statistical significance of the comparison between the two or three groups was analyzed using Student's t-test or one-way analysis of variance. Statistical significance was considered at *P* < 0.05.

## Results

### Hypoxia enhanced resistance to gemcitabine and stemness of PC cells

**Figure 1:**
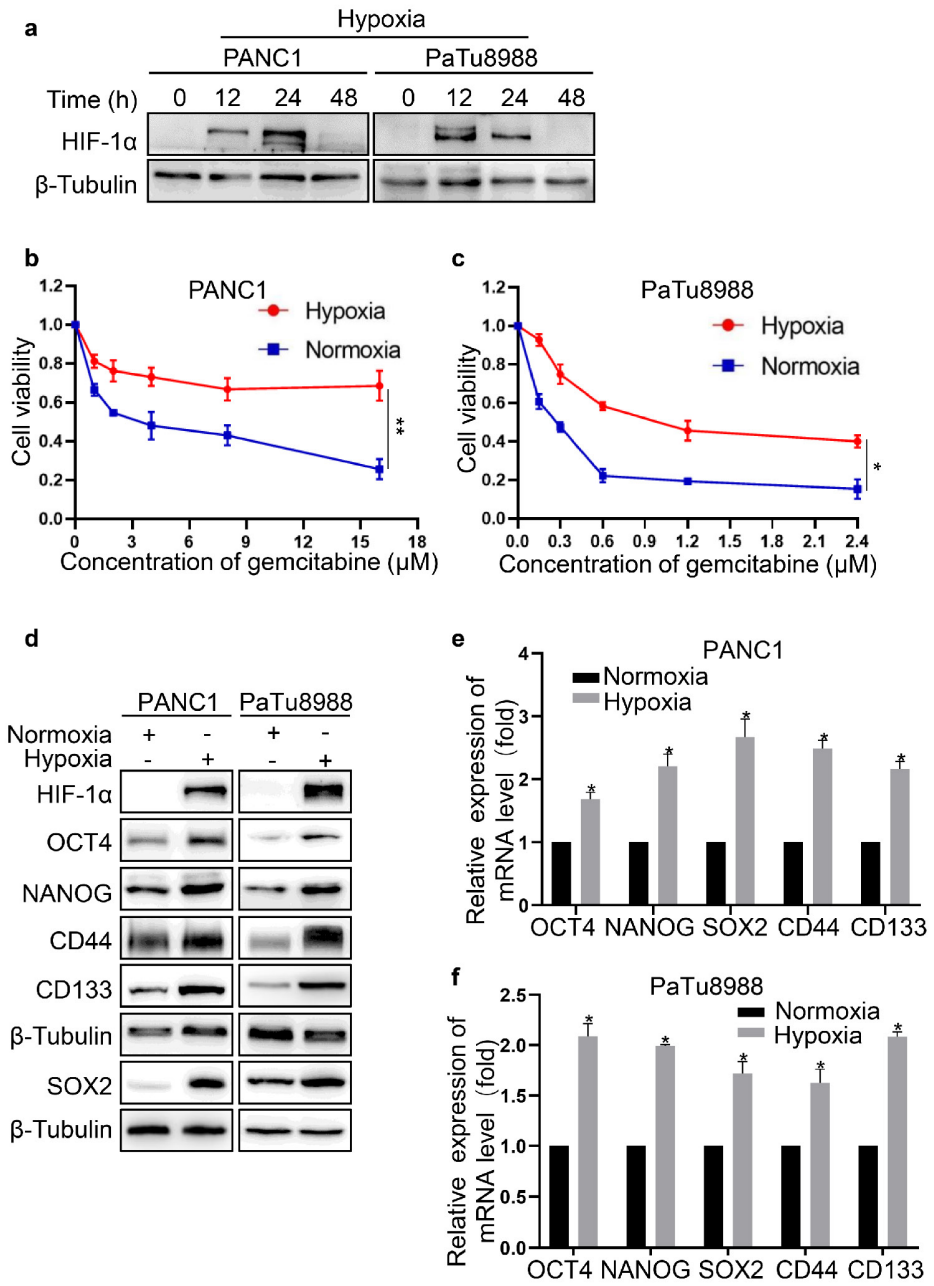
Hypoxia promoted the stemness and gemcitabine resistance of PC cells. (a) Western blotting was used to assess the expression level of HIF-1α in pancreatic cancer cells upon hypoxia. (b, c) Cell viability of normoxia or hypoxia groups was calculated by CCK-8 assay. (d) The protein level of stemness markers was detected in pancreatic cancer cells upon normoxia or hypoxia. (e, f) The mRNA level of stemness markers in pancreatic cancer cells upon normoxia or hypoxia was measured using qRT-PCR. Data were presented as mean±SD of three independent experiments. (*P <0.05, **P <0.01).

To investigate the effects of hypoxia, PANC1 and PaTu8988 cells were exposed to 1% O_2_ for 0, 12, 24 and 48 h. As shown in Fig. 1a, HIF-1α expression was significantly increased in PC cells for 12 h and 24 h of hypoxia compared with normoxia, confirming the effectiveness of hypoxic culture conditions. Moreover, the expression of HIF-1α was markedly decreased by 48 h (Fig. 1a). Thus, we selected 24 h of hypoxia for further experiments. The viability of PC cells was significantly decreased after treatment with gemcitabine at different concentration for 72 h (Fig. 1b, c). It seemed that PaTu8988 cells were more sensitive to gemcitabine compared with PANC1 cells (Fig. 1b, c). In addition, cell viability was significantly increased under hypoxia compared with normoxia, indicating that hypoxia enhanced resistance to gemcitabine in PC cells. Subsequently, the expression of stemness markers was examined by western blot. The expression of OCT4, NANOG, SOX2, CD44 and CD133 was raised at both mRNA and protein level (Fig. 1d-f), suggesting that hypoxia promoted the stemness of PC cells.

### Exosomes were internalized by PC cells

**Figure 2:**
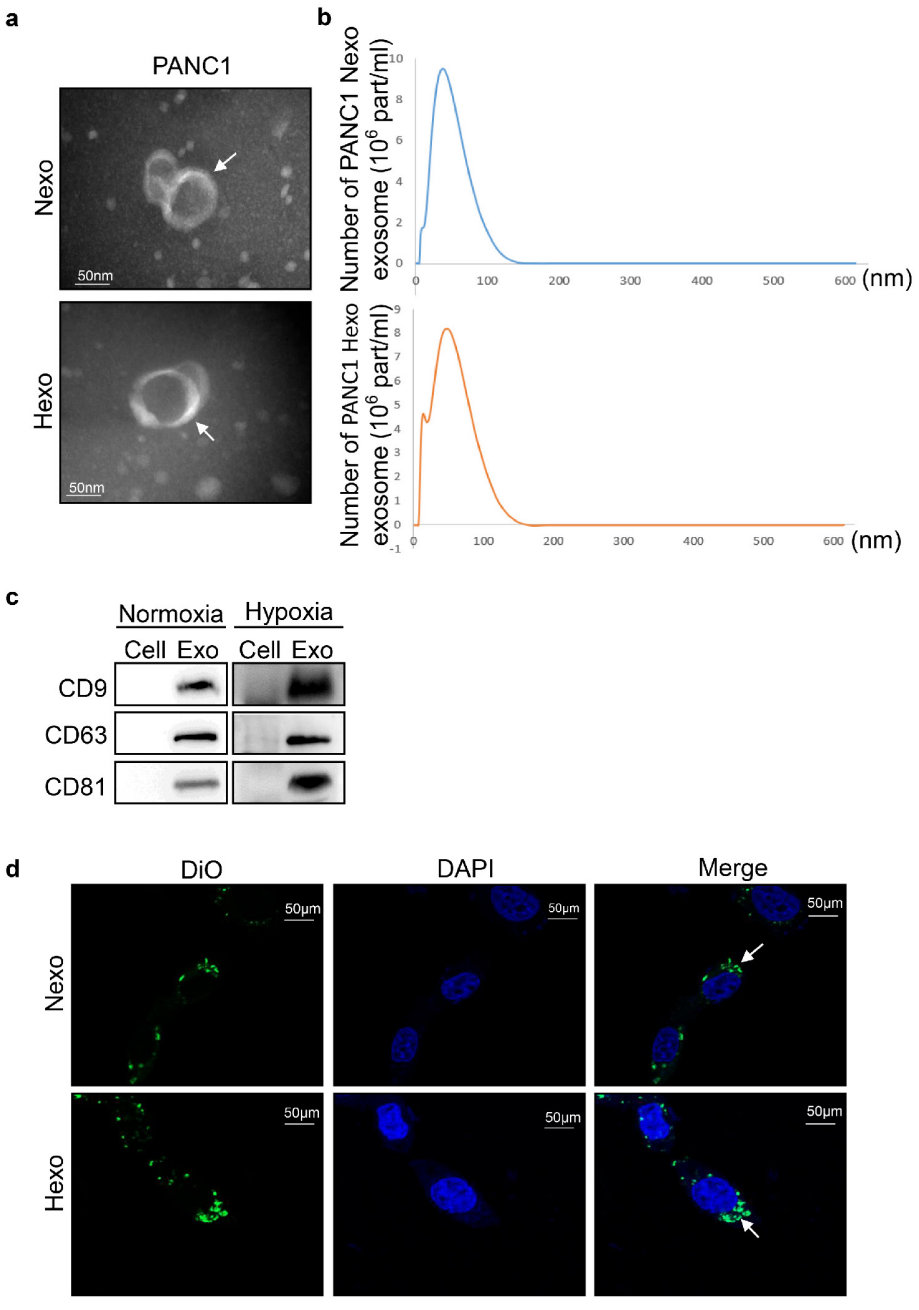
Exosomes were internalized by PC cells. (a) TEM images of exosomes isolated from cancer cells upon normoxia and hypoxia. Scale bar, 50 nm. (b) Nanoparticle tracking analysis of the size distribution and number of exosomes derived from PANC-1 cells isolated by ultracentrifugation. (c) Western blotting analysis of exosome markers CD9, CD63 and CD81 in exosomes (Exo) and cells upon PANC-1 normoxia or hypoxia. (d) Uptake of fluorescence (DiO)-labeled exosomes in PANC1 cells was quantified by confocal laser scanning microscope. The higher uptake was for Hexo. Scale bar, 50 μm.

Due to the PANC1 cells were less sensitive to gemcitabine than PaTu8988, we isolated exosomes from the supernatant of PANC1 cells and the exosomes were quantified by TEM and NTA. Round, cup-shaped particles (diameter: 40-150 nm) were exhibited by TEM, and a similar size distribution of exosomes was shown by NTA (Fig. 2a, b). The above results indicated that the exosomes were successfully isolated from the supernatant. Additionally, characteristic exosomal proteins CD9, CD63, and CD81 were expressed in both exosome types, and their levels were higher in Hexo versus Nexo (​Fig. 2c). To explore whether the exosomes were internalized by PANC1 cells, we incubated Dio-stained exosomes with PANC1 cells for 48 h. Confocal imaging confirmed that the exosomes were effectively internalized by PANC1 cells, and the internalization was higher for Hexo than Nexo (Fig. 2d). Thus, we concluded that the isolated exosomes could be internalized by PANC1 cells.

### Hexo promoted resistance to gemcitabine, proliferation, and stemness of PC cells

**Figure 3:**
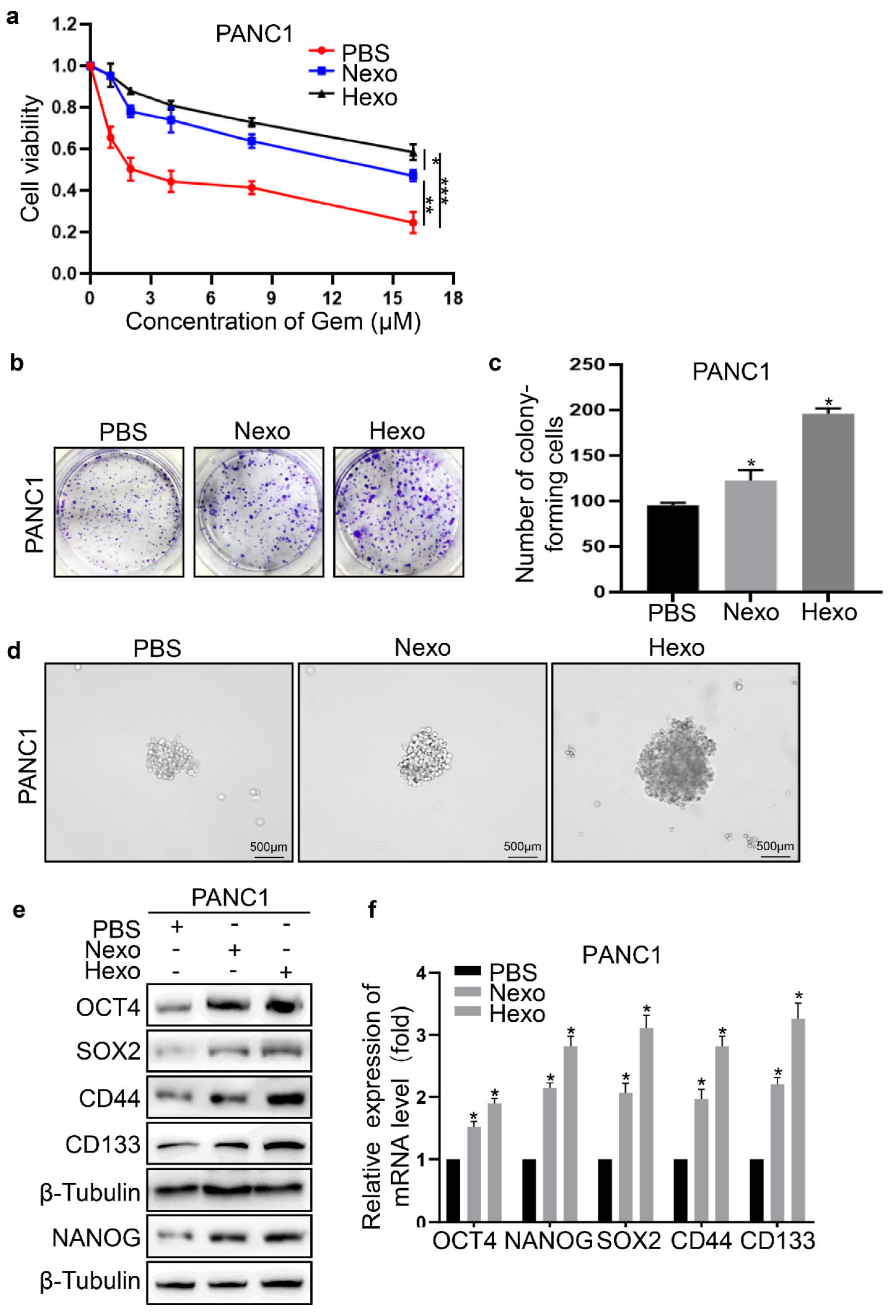
Hexo promoted the proliferation, stemness and gemcitabine resistance of PC cells. (a) Cell viability of PBS, Nexo or Hexo groups was calculated by CCK-8 assay. (b, c) Colony formation ability of PANC-1 cells treated with PBS, Nexo and Hexo was determined by colony formation assays. (d) The stemness of PANC1 was measured by sphere formation assays upon PBS, Nexo or Hexo. Scale bar, 500 μm. (e) Western blotting was used to assess the protein level of stemness markers in PANC1 cells upon PBS, Nexo or Hexo. (f) qRT-PCR was used to measure the mRNA level of stemness markers in PANC1 cells upon PBS, Nexo or Hexo. (*P<0.05, **P<0.01, *** P<0.001).

To explore whether Hexo affected PC cells, we investigated the resistance to gemcitabine, proliferation, and stemness of these cells after co-culture with exosomes. Compared with the PBS group, the viability of PANC1 cells in the exosome groups (i.e., Nexo and Hexo) was significantly enhanced by treatment with different concentrations of the drug (​Fig. 3a). The results indicated that tumor-derived exosomes exhibited obvious drug resistance. Moreover, the Hexo group exhibited stronger resistance to gemcitabine than the Nexo group, suggesting that exosomes secreted by PC cells under hypoxia reduced the sensitivity of normoxic PC cells to gemcitabine. Furthermore, the exosome groups formed more and larger clones than the PBS group (Figure 3B). The number of clones in the PBS, Nexo, and Hexo groups was 92 ± 6, 121 ± 17, and 194 ± 11, respectively (Fig. 3b, c). The results showed that Nexo and Hexo can promote the proliferation of PC cells, and Hexo play a more important role in promoting cell proliferation than Nexo. Next, we explored changes in the stemness of PC cells treated with tumor-derived exosomes. The ability for sphere formation was significantly enhanced in the Nexo and Hexo groups versus the PBS group (Fig. 3d). Moreover, the expression of cell self-renewal markers (SOX2, OCT4, and NANOG) and stem cell markers (CD44 and CD133) was increased in the Nexo and Hexo groups compared with the PBS group, both at the mRNA and protein levels (Fig. 3e, f). This evidence indicated that Hexo promoted the stemness of PC cells.

### Hexo antagonized gemcitabine-induced apoptosis in PC cells

**Figure 4:**
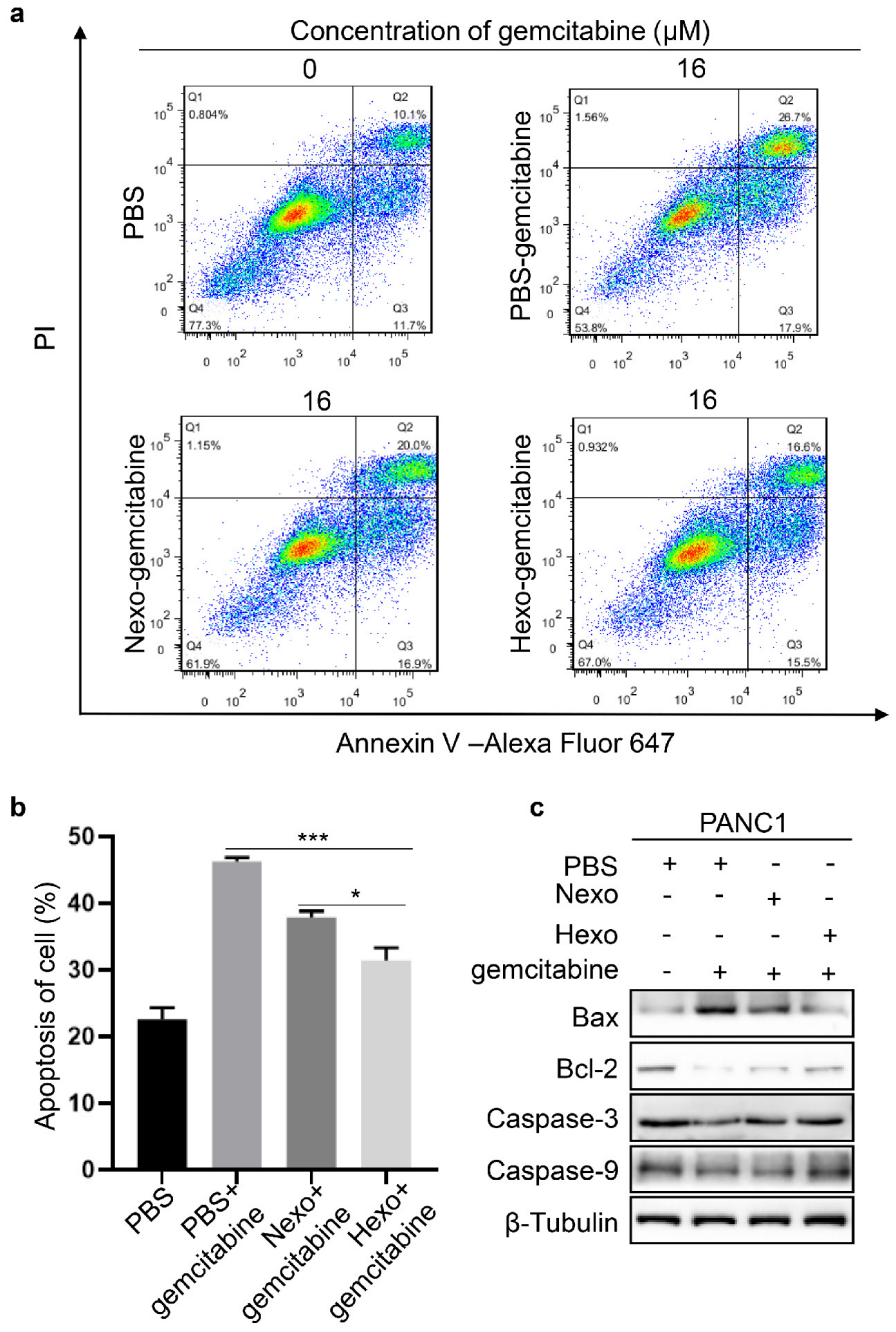
Hexo antagonized gemcitabine-induced apoptosis in PC cells. (a, b) Cell apoptosis was analyzed by Annexin V/PI apoptosis detection kit. (*P <0.05, **P<0.01, *** P<0.001) (c) The protein level of apoptosis markers was assessed by western blotting in PANC1 cells upon PBS, PBS-gemcitabine, Nexo-gemcitabine or Hexo-gemcitabine.

To detect the apoptosis of PANC1 cells, we performed flow cytometry in the PBS, PBS-gemcitabine, Nexo-gemcitabine, and Hexo-gemcitabine groups. The results (Fig. 4a, b) showed that the apoptotic rate was increased by approximately 16.6% in the PBS-gemcitabine group compared with the PBS group. Moreover, the rate of apoptosis in the Nexo-gemcitabine group was approximately 6.7% lower than that of the PBS-gemcitabine group. Furthermore, the apoptotic rate in the Hexo group was 10.1% lower and 6.5% higher than that detected in the PBS-gemcitabine group and the PBS group, respectively. In addition, in the Hexo-gemcitabine group, the expression of apoptosis proteins (Bax, CASP3, and CASP9) was decreased, whereas that of anti-apoptosis marker Bcl-2 was increased (Fig. 4c). The above results demonstrated that both Nexo and Hexo could inhibit gemcitabine-induced apoptosis of PC cells, and Hexo exerted a more obvious antagonizing effect on gemcitabine-induced apoptosis.

### Hexo inhibited gemcitabine-induced cell cycle arrest in PC cells

**Figure 5:**
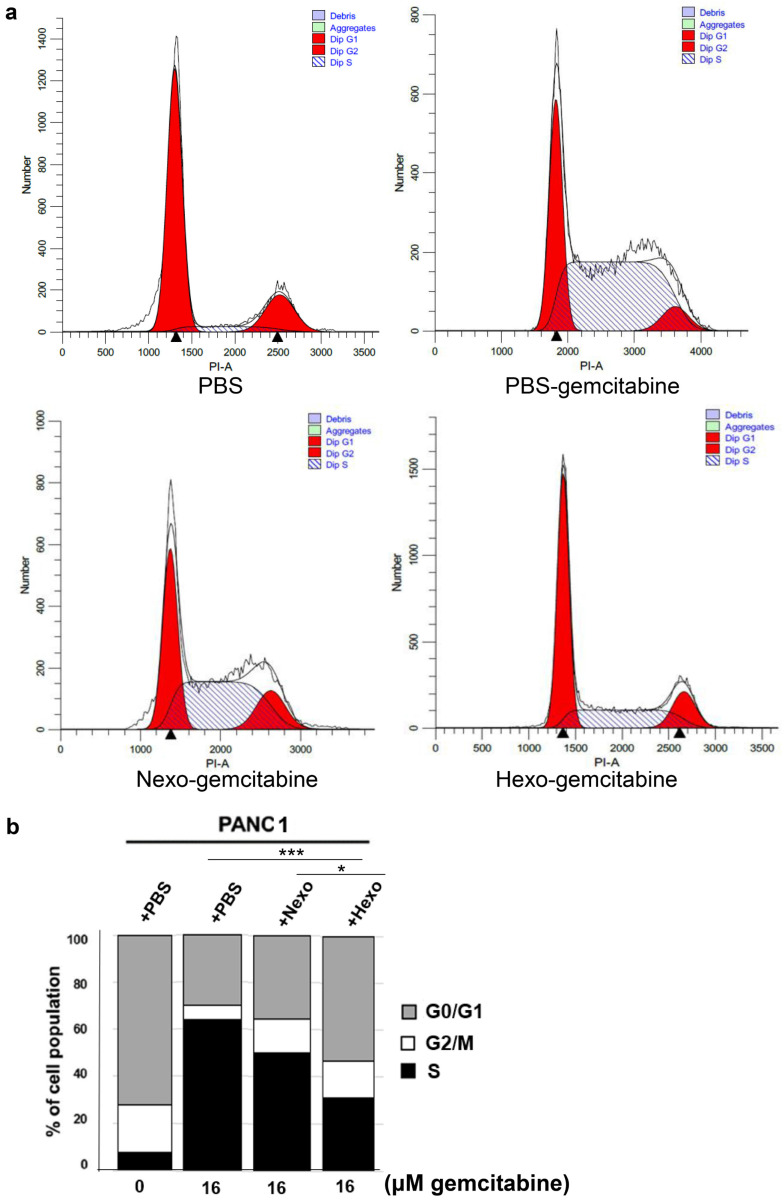
Hexo slowed down Gem-induced cell cycle arrest in PC cells. (a, b) Cell cycle profiles assessed by flow cytometry of PANC1 cells upon PBS, PBS-gemcitabine, Nexo-gemcitabine or Hexo-gemcitabine. (*P <0.05, *** P<0.001)

Using flow cytometry, we detected changes in the cell cycle to investigate the effect of Hexo on the cell cycle distribution of PC cells. We found that the proportion of cells in the S phase was gradually decreased in the PBS-gemcitabine, Nexo-gemcitabine, and Hexo-gemcitabine groups (63.7%, 49.97%, and 30.94%, respectively), whereas that of cells in the G2/M phase was increased (Fig. 5a, b). Compared with the PBS group, the number of cells in the S phase was significantly increased after treatment with 16 μM gemcitabine for 72 h, whereas the number of cells in the G2/M phase was markedly reduced (Fig. 5a, b). The results indicated that gemcitabine prevented the transition from S to G2/M phase of the cell cycle. Additionally, the proportion of cells in the S phase in the Hexo-gemcitabine group remained higher than that detected in the PBS group due to the action of gemcitabine. The above results suggested that tumor-derived exosomes could inhibit gemcitabine-induced cell cycle arrest in the S phase, and the effect of Hexo was stronger than that of Nexo.

### Hypoxia induced tumor-derived exosomal lncROR inhibited the activation of the Hippo/Yes-associated protein (Hippo/YAP) pathway in PC cells

**Figure 6:**
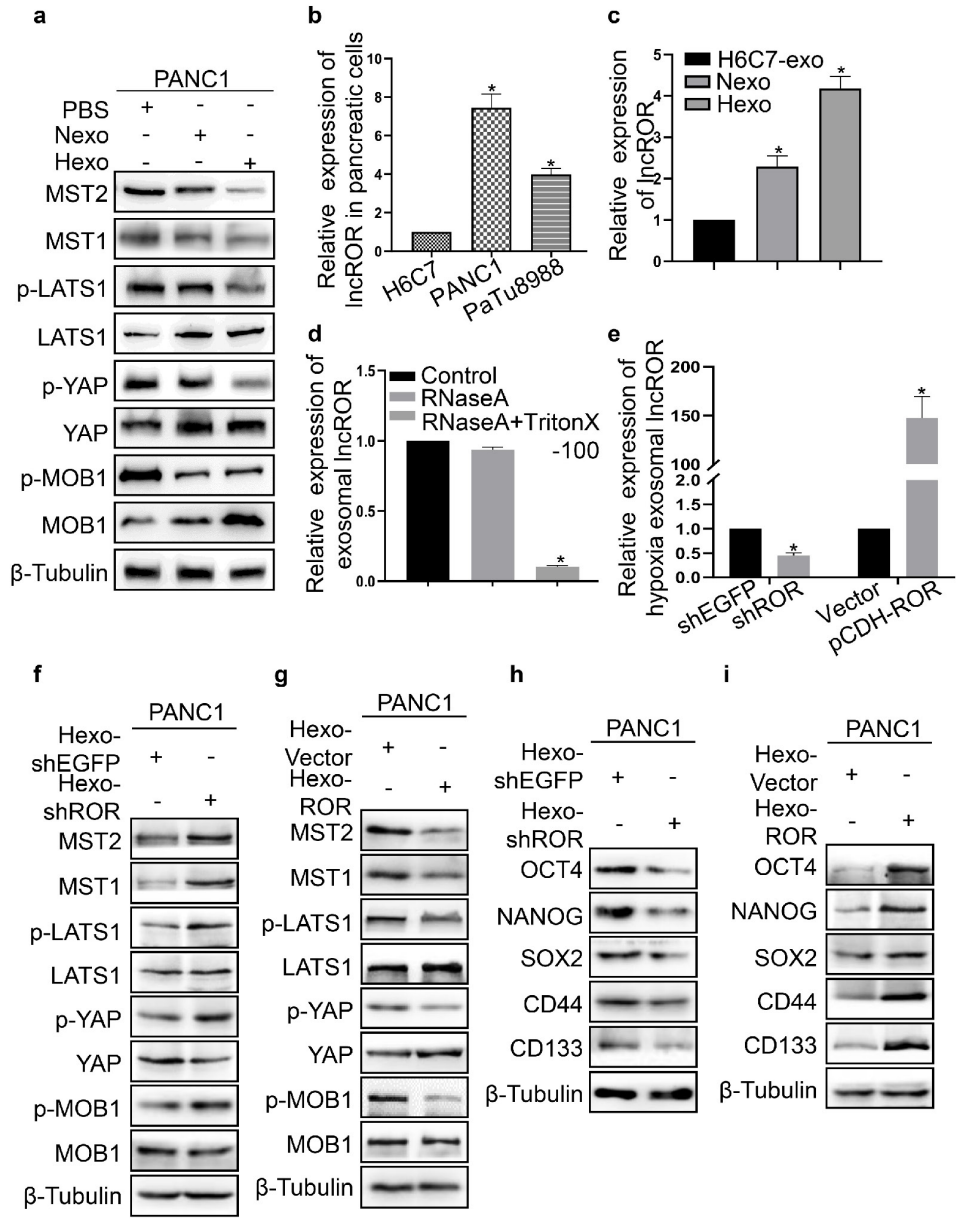
Hypoxia induced tumor-derived exosomal linc-ROR inhibited the Hippo pathway activation in PC cells. (a) The protein level of Hippo pathway was detected in PANC1 cells upon PBS, Nexo or Hexo. (b) Linc-ROR expression was measured in H6C7, PANC-1 and PaTu8988 cells using qRT-PCR. (c) qRT-PCR analysis of linc-ROR expression in the H6C7-exosomes (H6C7-exo), Nexo and Hexo. (d) The expression of linc-ROR was examined by qRT-PCR in exosomes Control, RNaseA (10 μg/ml), and RNaseA (10 μg/ml) + 0.3% Triton X-100 groups. (e) The expression of hypxia exosomal linc-ROR was tested after respectively transfection with shEGFP, shROR, Vector and pCDH-ROR. (f, g) Western blot was to determine hippo pathway proteins expression in PANC1 cells cultured with Hexo that transfected shEGFP, shROR, pCDH-Vector or pCDH-ROR. (h, i) Western blot was to determine stemness proteins expression in PANC1 cells cultured with Hexo that transfected shEGFP, shROR, pCDH-Vector or pCDH-ROR.

The Hippo pathway plays key roles in cell differentiation, proliferation, apoptosis, and regeneration 19. Therefore, we examined the expression of Hippo signaling proteins in the PBS, Nexo, and Hexo groups. Compared with the PBS group, the expression of YAP, Mps one binder 1 (MOB1), and large tumor suppressor kinase 1 (LATS1) in PANC1 cells was increased in the Nexo and Hexo groups. In contrast, the protein expression of phosphorylated-YAP (p-YAP), p-MOB1, p-LATS1, macrophage stimulating 1 (MST1), and MST2 was decreased (Fig. 6a). These findings indicated that Hexo can inhibit the activation of Hippo/YAP signaling to enhance chemoresistance in normoxic PC cells. According to a previous study, lncROR is vital for Hippo pathway inactivation 20. In this study, we determined the expression of lncROR in H6C7, PANC1, and PaTu8988 cells. The highest lncROR expression was observed in PANC1 cells, followed by PaTu8988 and H6C7 cells (Fig. 6b). Next, we examined the expression of lncROR in exosomes isolated from H6C7 and PANC1 cells. The levels of lncROR in Hexo or Nexo from PANC1 were higher than those measured in exosomes from H6C7 cells (Fig. 6c). The variation in lncROR expression was consistent with the change in protein expression in the Hippo pathway after treatment with exosomes. Therefore, we supposed that tumor-derived exosomes affected the resistance to gemcitabine and stemness of PC cells through the transfer of lncROR. Subsequently, the stability of exosomal lncROR was investigated. We measured lncROR expression in exosomes treated with RNase A (10 μg/mL) for 0 and 30 min. Upon RNase A digestion, there were no statistically significant changes in lncROR expression (Fig. 6d). However, exosomal lncROR expression was markedly reduced following co-treatment with RNase A and Triton X-100 (Fig. 6d). These data suggested that lncROR released by PC cells was wrapped in a double-layer membrane. Next, we investigated the role of exosomal lncROR in PANC1 cells. Downregulation and upregulation of lncROR expression in PANC1 cells led to a decrease and increase in hypoxic exosomal lncROR, respectively (Fig. 6e). Thereafter, the expression of Hippo signaling and stemness markers was detected in PANC1 cells co-cultured with Hexo-shEGFP, Hexo-shROR, Hexo-Vector, and Hexo-ROR, respectively. Following the downregulation of ROR, the expression of p-YAP, p-MOB1, p-LATS1, MST1, and MST2 was increased, whereas that of YAP, MOB1, and LATS1 was decreased (Fig. 6f). In contrast, upon upregulation of ROR, the expression of Hippo pathway proteins exhibited the opposite pattern (Fig. 6g). In addition, the expression of OCT4, NANOG, SOX2, CD44, and CD133 was reduced in the Hexo-shROR group (Fig. 6h). However, the expression of stemness markers was increased in the Hexo-ROR group (Fig. 6i). The above results indicated that Hexo promoted the stemness and chemoresistance of PC cells by transferring lncROR via Hippo signaling.

## Discussion

The present study demonstrated that hypoxia promoted stemness and induce resistance to gemcitabine in PC cells. Hypoxia stimulated PC cells to secrete more exosomes, and Nexo and Hexo could be transferred into PC cells. Thereafter, it was demonstrated that Hexo promoted the resistance to gemcitabine and stemness of PC cells. In addition, Hexo inhibited apoptosis and cell cycle arrest in PC cells. Furthermore, it was verified that Hexo inactivated the Hippo/YAP signaling pathway in PC cells by transferring exosomal lncROR.

Hypoxia is common in solid tumors, such as PC. This is attributed to the rapid growth of tumor cells, which requires the consumption of oxygen. The activation of HIF-1α drives vascular endothelial growth factor (VEGF) to promote vascular remodeling 21, thereby generating immature and hyperpermeable tumor-associated vasculature 22. These effects lead to insufficient tumor perfusion that exacerbates hypoxia, forming a vicious circle. Several studies found that hypoxia promoted stemness and secondary chemotherapy resistance through the activation of HIFs and the hypoxia cascade 23, 24. Xia et al. discovered that sorafenib-induced hypoxia and expression of the HIF family caused metabolic and microenvironmental changes, and played a role in regulating stemness, epithelial-mesenchymal transition, metabolic reprogramming, angiogenesis, immune inhibition, and sensitivity to sorafenib 25. Tang et al. found that the hypoxic TME promoted stemness and chemoresistance in colorectal cancer 26. In this study, we found that hypoxia increased the expression of HIF-1α and stemness-related markers. In addition, hypoxia weakened the sensitivity of PC cells to gemcitabine.

Exosomes secreted by tumor cells and stromal cells are key mediators of cell-to-cell communication in the TME and widely involved in the induction of drug resistance. Hu et al. confirmed that miR-92a-3p in exosomes secreted by tumor-associated fibroblasts promoted stemness and resistance in colorectal cancer cells by inhibiting F-box and WD repeat domain containing 7 (FBXW7) and modulator of apoptosis 1 (MOAP1) 27. Additionally, exosomes secreted from cancer-associated fibroblasts induced drug resistance in malignant lymphoma 28. Exosomes secreted by tumor cells and stromal cells can transmit signaling molecules to promote the development of drug resistance. The hypoxic TME stimulates the secretion of large amounts of exosomes by tumor cells 29. Therefore, we investigated whether exosomes secreted by PC cells in the hypoxic TME could induce resistance to gemcitabine in normoxic PC cells. Considering that PANC1 cells were more resistant to gemcitabine than PaTu8988 cells, we extracted exosomes secreted by PANC1 cells under normoxia and hypoxia. Our analysis confirmed that these tumor-derived exosomes could be endocytosed by recipient cells. Next, we demonstrated that tumor-derived exosomes promoted resistance to gemcitabine and stemness of PC cells, and the effects of Hexo were more pronounced than those of Nexo. The above results confirmed that Hexo could induce drug resistance to normoxic tumor cells.

Gemcitabine, a deoxycytidine analog, enters the cytoplasm through a nucleoside transporter and is phosphorylated by the rate-limiting enzyme deoxycytidine kinase (DCK) to generate gemcitabine monophosphate 30. The final metabolite, gemcitabine triphosphate, is incorporated into normal DNA double strands to inhibit DNA synthesis, thereby inducing apoptosis 30. Therefore, we used flow cytometry to investigate cell apoptosis, and found that it was significantly increased upon treatment of PANC1 cells with gemcitabine. However, the combination of gemcitabine with Nexo or Hexo reduced the apoptosis of PC cells, indicating that Hexo could antagonize gemcitabine-induced apoptosis in these cells.

The cell cycle is a process by which mammalian cells regulate proliferation. It involves four stages: DNA replication occurs in the S phase; in the M phase (mitosis), DNA and cellular components divide to form two daughter cells; the G2 phase occurs between the S and M phases, during which cells prepare for mitosis; and the G1 phase occurs after mitosis and before the S phase, in which cells prepare for another round of DNA and cell replication 31. Normal DNA synthesis is disrupted by treatment with gemcitabine, thereby irreversibly inhibiting ribonucleotide reductase and inducing cell cycle arrest in the S phase 31. The results of this study showed that gemcitabine could induce S-phase arrest in PANC1 cells. Compared with the PBS-gemcitabine group, the S-phase arrest induced by gemcitabine was attenuated in the exosome-treatment group, particularly Hexo. These results indicated that Hexo could inhibit the cell cycle arrest induced by gemcitabine.

We revealed that Hexo could transfer hypoxia-induced resistance to gemcitabine in normoxic PC cells, dedifferentiate them into a cancer stem-like state, and reduce their drug sensitivity to gemcitabine. However, the mechanism underlying these effects remains unclear. Recent studies showed that the Hippo pathway can regulate primary and acquired resistance to tumor therapy 32. Abnormal YAP activation in the Hippo pathway significantly reduced the efficacy of targeted therapy and enhanced resistance to epidermal growth factor receptor (EGFR) tyrosine kinase inhibitors in lung cancer 33. Furthermore, evidence showed that the Hippo pathway was involved in glioma progression and activation of chemoresistance mechanisms 34. Dysregulation of the YAP/transcriptional coactivator with PDZ-binding motif (YAP/TAZ) signaling may be an important mechanism of intrinsic and acquired resistance to targeted therapies and chemotherapy 35. In this study, we found that Hexo inhibited the activation of the Hippo pathway in PC cells. A previous study reported that lncROR promoted the localization of YAP into the nucleus to inactivate the Hippo pathway in PC cells 20. Consequently, we hypothesized that Hexo promoted stemness and chemoresistance of PC cells by transferring lncROR via Hippo signaling. Next, we demonstrated that the levels of lncROR were higher in the chemoresistant PANC1 cells. As expected, hypoxia induced lncROR expression in exosomes. Furthermore, exosomal lncROR promoted stemness and inactivated Hippo signaling in PC cells.

## Conclusion

In summary, the present findings demonstrated that Hexo promoted stemness and induced resistance to gemcitabine in PC cells. Moreover, Hexo antagonized gemcitabine-induced apoptosis and inhibited gemcitabine-induced cell cycle arrest in PC cells. Mechanistically, Hexo promoted stemness to enhance chemoresistance in PC cells by transferring lncROR via Hippo signaling. The results of this study suggested that exosomal lncROR may serve as a candidate target of chemotherapy for PC.

## Figures and Tables

**Table 1:** DNA and RNA nucleotide sequences

U6-F	CTCGCTTCGGCAGCACA
U6-R	AACGCTTCACGAATTTGCGT
ROR-F	TGCTCCGTGAGAAAGATCCA
ROR-R	GCCGCTAAGCCAAGAAGATC
OCT4-F	CTTGAATCCCGAATGGAAAGGG
OCT4-R	CCTTCCCAAATAGAACCCCCA
NANOG-F	CCCCAGCCTTTACTCTTCCTA
NANOG-R	CCAGGTTGAATTGTTCCAGGTC
SOX2-F	TACAGCATGTCCTACTCGCAG
SOX2-R	GAGGAAGAGGTAACCACAGGG
CD44-F	CTGCCGCTTTGCAGGTGTA
CD44-R	CATTGTGGGCAAGGTGCTATT
CD133-F	GGCCCAGTACAACACTACCAA
CD133-R	ATTCCGCCTCCTAGCACTGAA
GAPDH-F	TGGGGAAGGTGAAGGTCGG
GAPDH-R	CTGGAAGATGGTGATGGGA
shEGFP-F	CCGGTACAACAGCCACAACGTCTATCTCGAGATAGACGTTGTGGCTGTTGTATTTTTG
shEGFP-R	AATTCAAAAATACAACAGCCACAACGTCTATCTCGAGATAGACGTTGTGGCTGTTGTA

## Abbreviations

PC: Pancreatic cancer
TME: Tumor microenvironment
HIF-1α: Hypoxia-inducible factor 1α
Hexo: Hypoxia-induced tumor-derived exosomes
CCK-8: Cell Counting Kit 8
qRT-PCR: Quantitative real-time polymerase chain reaction
Nexo: Normoxia exosomes
VEGF: Vascular endothelial growth factor
HIFs: Hypoxia-inducible factors
dCK: Deoxycytidine kinase
EGFR: Epidermal growth factor receptor
TKIs: Tyrosine kinase inhibitors
